# Sociodemographic Factors Influenced Response to the 2015 National Nutrition Survey on Preschool Children: Results From Linkage With the Comprehensive Survey of Living Conditions

**DOI:** 10.2188/jea.JE20180176

**Published:** 2020-02-05

**Authors:** Hitomi Okubo, Tetsuji Yokoyama

**Affiliations:** Department of Health Promotion, National Institute of Public Health, Saitama, Japan

**Keywords:** response rates, sociodemographic factors, multiple imputation, national surveys, Japan

## Abstract

**Background:**

The National Nutrition Survey on Preschool Children, Japan (NNSPC) provides fundamental information for policy making for child nutrition. However, the response rate and background characteristics of subjects are unclear. Here, we examined response rate and sociodemographic factors related with response to the survey and evaluated the magnitude of bias due to selective response in the survey estimates of the NNSPC.

**Methods:**

This study was based on two national surveys conducted in 2015: the NNSPC and the Comprehensive Survey of Living Conditions (CSLC). Because potential survey participants of the NNSPC were children aged <6 years and their households that answered the CSLC, we examined response rates and respondent characteristics by linking the data of the NNSPC and CSLC. Multiple logistic regression analysis was used to identify sociodemographic factors associated with response. Potential bias caused by non-response in the survey estimates was examined after considering missingness through multiple imputation.

**Results:**

Among the 5,343 children who participated in the CSLC, 3,426 children responded to the NNSPC (response rate = 64.1%). Variables associated with response were living in a smaller city, a large number of children, three-generation family structure, older maternal age, and a non-working mother. The prevalence of overweight was underestimated by 20%, but the bias for almost all variables examined was small.

**Conclusions:**

Response to the survey varied by sociodemographic characteristics. Some biases, mostly small, were seen in survey estimates of the 2015 NNSPC. Further insight into the effect of selective response is important to assess associations between variables more precisely.

## INTRODUCTION

It is now widely recognized that response rates in epidemiological studies have dramatically declined over the last few decades.^1^^–^^3^ Decreasing participation in survey research increases the risk of selection bias and errors in statistical inferences.^4^ Accumulating evidence of selection bias in health surveys has shown that participants differ from non-participants in several characteristics.^5^^–^^16^ In general, participants (respondents) are more likely to have a higher socioeconomic status (higher levels of education and income),^5^^–^^12^ a better health status,^5^^,^^9^ a healthier lifestyle,^5^^,^^9^^–^^13^ and a lower mortality rate for specific diseases than non-participants (non-respondents).^14^^–^^16^

The National Nutrition Survey on Preschool Children (NNSPC), a questionnaire survey conducted in Japan every 10 years since 1985, assesses the feeding practices, dietary intake, lifestyle, and health status of preschool children living in Japan and provides valuable information for nutrition policy development.^17^ The survey findings have been used in the planning and promotion of breastfeeding and healthy diets for young Japanese children. However, the response rate and background characteristics of respondents, which are important indicators of representativeness, have not been published in any official reports. Survey participants of the NNSPC are drawn from among infants and preschool children (aged <4 years in 1985, 1995, and 2005, and aged <6 years in 2015)^17^ and their households who had answered another national survey, the Comprehensive Survey of Living Conditions (CSLC),^18^ conducted earlier in the same respective year. Linking data from the NNSPC and CSLC thus allows us to address response rates and the degree to which study participants differ from the total population. Understanding the background characteristics of respondents in the national survey is critically important for policy making and subsequent research. It might, therefore, be helpful to consider the possibility of selection bias and the external validity of the survey findings and to establish survey strategies for subject sampling and data collection.

The aim of the present study was to examine the response rate and sociodemographic factors related with response to the 2015 NNSPC by linking data with the CSLC. Additionally, we evaluated the magnitude of bias in the survey statistics obtained from the 2015 NNSPC due to selective response using a multiple imputation approach to account for mssingness.^19^

## METHODS

### Data of two national surveys

This study was based on data from two national surveys conducted in 2015 by the Japanese Ministry of Health, Labour and Welfare (MHLW): the CSLC^20^ and the NNSPC.^21^ Data from the two surveys were used with permission from the MHLW. The CSLC has collected comprehensive information on the living conditions of people in Japan, such as socio-demographics, health, medical care, welfare, and income, since 1986. The CSLC is conducted as a large-scale survey every 3 years and a small-scale survey in each interim year.^18^ The 2015 small-scale survey consisted of two surveys of household and income questionnaires.^20^ The household questionnaire covered 59,425 households and household members who lived in 1,106 districts randomly sampled from the National Census in 2010.^22^ The income questionnaire covered 9,036 and household members in 500 districts randomly selected from these 1,106 districts. The two questionnaires were distributed to potential eligible households in advance of each survey date (on June 4^th^ for the household questionnaire and July 16^th^ for the income questionnaire), and later collected by trained investigators during home visits.^20^ Of the 46,651 households that answered the household questionnaire (response rate = 78.5%),^20^ data from 46,634 households (115,941 household members) for the household questionnaire and from 6,706 households (17,219 household members) for the income questionnaire were provided by the MHLW after excluding unclear answers.

The NNSPC has been conducted every 10 years since 1985 on the basis of the Statistics Act (General Statistical Surveys, Articles 19 to 23)^23^ to assess methods of feeding in infancy and the diet and lifestyle of preschool children living in Japan and to obtain basic data required for the planning and promotion of breastfeeding and a healthy diet in early childhood.^17^ Potential participants of the NNSPC were children aged <6 years (born from 1st of June, 2009 to 31st of May, 2015) (approximately 5.5 thousand children) and their households (approximately 4.4 thousand children) in the 1,106 districts set for the 2015 CSLC.^21^ Of these, three districts in Ibaraki Prefecture were excluded from the survey in the aftermath of heavy rain which occurred in September 2015. Trained investigators visited each potential household a single time during September to distribute a self-administered questionnaire for each child to the mother or guardian, who was usually responsible for food preparation, and later collected information about breastfeeding, weaning, introduction of solid foods, food allergy, child’s health status and lifestyle, parental lifestyle, and basic characteristics of the family. Of the 2,992 households (3,936 children aged <6 years) who answered the questionnaire,^21^ data from 2,950 households (3,871 children) were provided by the MHLW after excluding those with unclear answers about child’s age.

These surveys were conducted according to the guidelines of the Declaration of Helsinki, and verbal informed consent was obtained from all study participants or their guardian. The CSLC and NNSPC are both conducted by the MHLW, Japan, and have stringent protocols and procedures that ensure confidentiality and protect individual participants from being identified. Additionally, the present secondary analysis was based on a public-use dataset consisting solely of information that had already been anonymized. Accordingly, Institutional Review Board approval was not required.

### Data linkage

As the potential study participants of the 2015 NNSPC were children aged <6 years and their households that answered the 2015 CSLC,^20^ we examined the characteristics of participants in the NNSPC by linking its data with those of the CSLC. We initially linked the two databases at the household level using information on prefecture, area, unit block, and household number that was common to both surveys. Among the 2,950 households of the 2015 NNSPC, 2,876 households (97.5%) were linked to the 2015 CSLC. However, the 2015 CSLC answers of 80 of these 2,876 households revealed that they had no children aged <6 years, which meant that some data were linked incorrectly. In particular, 55.0% of these mislinked households were located in one prefecture. Careful review revealed that the household number assigned for the CSLC in this prefecture was not utilized in the NNSPC. To increase the accuracy of data linkage, we refrained from linkage at the household level and instead linked the two databases at the individual level.

**Figure 1.**
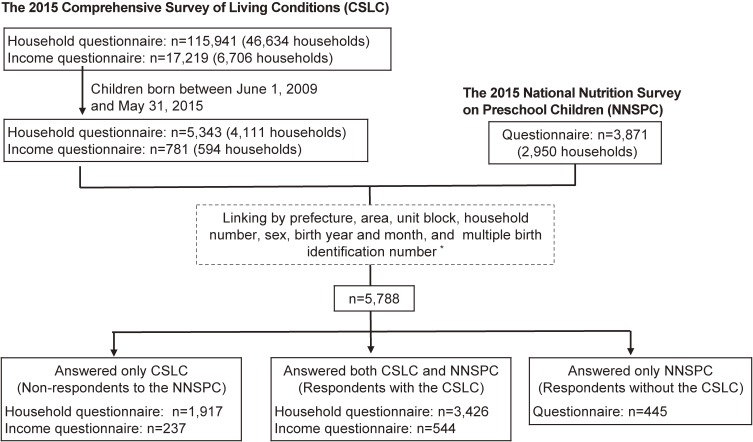
Flow chart of respondents in the 2015 NNSPC. ^a^One of 47 prefectures did not use the same household number in the CSLC and NNSPC. We therefore used information on other variables apart from household number for data linkage as an exception.

Among the 115,941 participants of the 2015 CSLC, we initially restricted to the 5,343 children who were born from the 1st of June, 2009 to the 31st of May, 2015 (Figure 1). Information to identify each participant within the household was contained in the CSLC, but not in the NNSPC. Therefore, we linked the two databases at the individual level using the information on sex and birth year and month, in addition to the variables described above. Because there were some cases of multiple births (47 twins and 1 triplets), we also used information to identify multiple births within the households, which was newly created for the present study in consideration of birth order. For the one prefecture that did not use the same household number in both surveys, we used information on other variables apart from household number as an exception, and later confirmed that 60 individuals were additionally linked. Among the 5,788 children who answered either or both the CSLC and NNSPC, 1,917 children had data for the CSLC only, 445 children had data for the NNSPC only and 3,426 children had both. For the present study, we defined the 3,426 children who had both data and the 1,917 children who had data for the CSLC only as respondents and non-respondents to the NNSPC, respectively. Two authors independently conducted the data linkage and confirmed the consistency of the results.

### Assessment of sociodemographic and lifestyle characteristics

Information on sociodemographic and socioeconomic variables was explored from the 2015 CSLC.^20^ From the household questionnaire, we obtained information on child’s sex (boy or girl), child’s age as of May 31, 2015 (0–1, 2–3, and 4–5 years), household structure (parents and unmarried children only, single parent and unmarried children only, three-generation family, and others), number of unmarried children aged <18 years (1, 2, and ≥3 persons), maternal and paternal educational attainment (junior high school or less; high school; technical or professional school/college; and university or higher), maternal and paternal labour force status (regular staff/employee; non-regular staff/employee; executive of company/organization; self-employed/family worker/industrial homework; others; and non-worker), and household expenditure in May. Equivalent household expenditure was calculated by dividing household expenditure in May by the square root of household size^24^ and then categorizing the result into thirds (low, middle, and high). Maternal and paternal age categories were defined (<30, 30–39, and ≥40 years). Residential blocks were grouped into six regions (Hokkaido and Tohoku; Kanto; Hokuriku and Tokai; Kinki; Chugoku and Shikoku; and Kyushu). Residential areas were also grouped into four categories according to population size (metropolitan areas; city with population ≥150 thousand; city with population <150 thousand; and towns and villages; hereafter referred to as ‘size of residential area’). Information on total household annual income and self-assessed living conditions (very difficult, somewhat difficult, normal, somewhat comfortable, and very comfortable) was obtained from the income questionnaire. Equivalent household annual income was calculated by dividing total household annual income by the square root of household size, and then categorizing the result into thirds (low, middle, and high). Missing data for household expenditure, maternal and paternal age, educational attainment, and labor force status were categorized as missing.

### Statistical analysis

Response rates by sociodemographic variables were calculated using the number of participants aged <6 years in the 2015 CSLC as the denominator. The chi-square test was used to confirm homogeneity. Multivariate logistic regression analysis was performed, and the odds ratios (ORs) and 95% confidence intervals (CIs) were calculated as measures of the strength of the association between the response and potential explanatory factor of interest. These analyses were controlled for potential confounders, including child’s sex, child’s age, size of residential area, residential block, household structure, number of unmarried children aged <18 years, equivalent monthly household expenditure, maternal age, maternal educational attainment, maternal occupation, paternal age, paternal educational attainment, and paternal occupation. Of the 4,111 households (5,343 children) included in the analysis, 1,117 households had at least two participating children. Therefore, we used robust standard errors to consider intraclass correlations among children in the same household. Some of the study participants answered the income questionnaire in the CSLC. We conducted a sub-analysis among subjects who answered the income questionnaire to examine the associations between economic characteristics (income level and self-assessed living conditions) and response to the NNSPC.

In the subsequent analysis, we examined the magnitude of potential bias in the survey estimates of the 2015 NNSPC due to missing data (non-response). There are two different sources of missing data in the NNSPC, the first attributable to respondents of the NNSPC who did not answer certain questions (item non-response) and the second to potential survey participants who did not respond to the 2015 NNSPC (survey non-response). We assumed that data were at least missing at random since the missing pattern was significantly determined by observed variables.^19^ We, therefore, used the multiple imputation procedure of the SAS statistical software (Proc MI; SAS Institute, Cary, NC, USA) to impute missing data and compared survey estimates of the NNSPC between data from respondents (*n* = 3,426) and those from potential survey participants (*n* = 5,343). Survey estimates selected here were child’s sex, child’s age as of May 31, 2015, birthweight and length, gestational age, current body weight and height, birth order, place of day care (eg, nurseries, kindergarten, certified centers for early childhood education and care, and so on), history of food allergy, bowel movements per week, wake-up time on weekdays, bedtime on weekdays, maternal age, maternal employment status, self-assessed economic condition, self-assessed time allowance, and self-assessed overall living conditions. These were selected because they were common question items observed in the 2015 NNSPC questionnaires for both 0–1-year-old and 2–5-year-old children. To deal with item non-response among the respondents, the variables of the 2015 NNSPC mentioned above were used for the imputation model. The imputation process was repeated to create five imputed datasets using the fully conditional specification. For each of the five imputed datasets, we further imputed data for the 1,917 non-respondents to the NNSPC to deal with survey non-response. The variables included in the imputation models were residential block, size of residential area, and household structure as well as the variables mentioned above. In total, 25 imputed datasets were created and the results were then averaged over these datasets using the MIANALYZE procedure of the SAS statistical software. To evaluate the magnitude of bias parameters due to missingness, we divided the difference in the survey estimates between respondents and potential survey participants after imputation by the imputed survey estimates in potential survey participants and multiplied by 100. As the NNSPC data might be used to examine not only for point estimates of population parameters but also associations between variables, we need to know the magnitude of potential bias in associations between targeted outcomes and certain sociodemographic variables that significantly influence the response to the survey. We also examined ORs of overweight, as an example of targeted outcome, by sociodemographic variables before and after imputation. Overweight was defined according to the age- and sex-specific BMI cut-offs for Japanese children using the LMS method, which corresponded to BMI of 25 kg/m^2^ at 17.5 years of age.^25^

All reported *P* values are two-tailed, and *P* < 0.05 was considered to be statistically significant. All statistical analyses were performed using SAS statistical software version 9.4.

## RESULTS

### Response rate

Of the 5,343 children aged <6 years who answered the household questionnaire of the 2015 CSLC, 3,426 children responded to the 2015 NNSPC. The overall response rate was 64.1%.

Table 1 shows the response rate according to sociodemographic characteristics. Differences in response rate were observed across categories of almost all sociodemographic characteristics except for child’s sex and age (all *P* < 0.05).

**Table 1.** Response rates and odds ratios for response to the 2015 National Nutrition Survey on Preschool Children, Japan, according to sociodemographic characteristics among children aged <6 years and their households

	Total	Respondents		*P* value^a^	Multivariate^b,c^	
	
		*n*	Response rate, %		OR	95% CI
Number	5,343	3,426	64.1			
Child’s sex						
Boys	2,731	1,753	64.2	0.92	1.00	(Reference)
Girls	2,612	1,673	64.1		0.96	(0.88–1.05)
Child’s age						
0–1 year	1,691	1,089	64.4	0.94	1.00	(Reference)
2–3 years	1,811	1,156	63.8		1.00	(0.92–1.10)
4–5 years	1,841	1,181	64.1		1.02	(0.91–1.13)
Size of residential area						
Metropolitan area	1,537	877	57.1	<0.001	1.00	(Reference)
City with population ≥150,000	1,654	1,079	65.2		1.40	(1.19–1.65)
City with population <150,000	1,741	1,192	68.5		1.56	(1.31–1.85)
Towns and villages	411	278	67.6		1.47	(1.18–1.94)
Residential blocks						
Hokkaido and Tohoku	488	295	60.5	<0.001	1.00	(Reference)
Kanto	1,940	1,185	61.1		1.01	(0.80–1.27)
Hokuriku and Tokai	896	631	70.4		1.40	(1.08–1.82)
Kinki	757	468	61.8		1.04	(0.80–1.36)
Chugoku and Shikoku	522	346	66.3		1.20	(0.90–1.61)
Kyushu	741	501	67.6		1.39	(1.06–1.83)
Household structure						
Parents and unmarried children only	4,326	2,778	64.2	<0.001	1.00	(Reference)
Single parent and unmarried children only	168	70	41.7		0.64	(0.39–1.06)
Three-generation family	736	511	69.4		1.35	(1.08–1.69)
Others	113	67	59.3		1.02	(0.61–1.69)
Number of unmarried children aged <18 years						
1 person	1,659	984	59.3	<0.001	1.00	(Reference)
2 people	2,429	1,589	65.4		1.20	(1.04–1.40)
≥3 people	1,255	853	68.0		1.24	(1.03–1.50)
Equivalent household expenditure						
Low (<100,000 Japanese yen/month)	1,632	1,049	64.3	0.01	1.00	(Reference)
Middle (100,000–139,999 Japanese yen/month)	1,840	1,221	66.4		1.09	(0.92–1.29)
High (≥140,000 Japanese yen/month)	1,770	1,101	62.2		0.97	(0.82–1.15)
Missing	101	55	54.5		0.69	(0.43–1.09)
Maternal age						
<30 years	1,037	624	60.2	0.02	1.00	(Reference)
30–39 years	3,365	2,190	65.1		1.20	(0.96–1.47)
≥40 years	908	594	65.4		1.35	(1.03–1.77)
Missing/children without mother	33	18	54.5		1.50	(0.40–5.61)
Maternal educational attainment						
Junior high school or less	206	121	58.7	<0.001	1.00	(Reference)
High school	1,636	1,035	63.3		1.10	(0.77–1.56)
Technical school or college	1,868	1,280	68.5		1.30	(0.91–1.87)
University or beyond	1,384	857	61.9		1.05	(0.72–1.53)
Missing/children without mother	249	133	53.4		1.08	(0.56–2.10)
Maternal labor force status						
Regular staff/employee	2,003	1,199	59.9	<0.001	1.00	(Reference)
Non-regular staff/employee	368	224	60.9		1.05	(0.81–1.35)
Executives of companies/organizations	36	28	77.8		2.28	(0.84–6.21)
Self-employed/family worker/industrial homework	329	232	70.5		1.30	(0.95–1.77)
Others	38	25	65.8		1.10	(0.51–2.34)
Non-workers	2,514	1,693	67.3		1.47	(1.27–1.70)
Missing/children without mother	55	25	45.5		0.70	(0.23–2.10)
Paternal age						
<30 years	664	419	63.1	<0.001	1.00	(Reference)
30–39 years	2,896	1,889	65.2		0.95	(0.74–1.22)
≥40 years	1,436	944	65.7		0.95	(0.71–1.26)
Missing/children without father	347	174	50.1		1.35	(0.42–4.31)
Paternal educational attainment						
Junior high school or less	268	164	61.2	<0.001	1.00	(Reference)
High school	1,585	1,045	65.9		1.08	(0.79–1.48)
Technical school or college	808	535	66.2		1.15	(0.82–1.62)
University or more	2,146	1,408	65.6		1.25	(0.90–1.74)
Missing/children without father	536	274	51.1		0.77	(0.39–1.53)
Paternal labor force status						
Regular staff/employee	4,112	2,665	64.8	<0.001	1.00	(Reference)
Non-regular staff/employee	73	44	60.3		0.94	(0.57–1.55)
Executives of companies/organizations	227	155	68.3		1.04	(0.73–1.48)
Self-employed/family worker/industrial homework	469	314	67.0		1.04	(0.80–1.34)
Others	37	29	78.4		1.49	(0.61–3.67)
Non-workers	65	40	61.5		0.90	(0.50–1.59)
Missing/children without father	360	179	49.7		0.79	(0.27–2.26)

CI, confidence interval; OR, odds ratio.

^a^The chi-square test was used to confirm homogeneity.

^b^Considered intraclass correlations among children in the same household.

^c^Adjusted for all of the variables listed in the Table.

### Association between sociodemographic factors and response to the 2015 NNSPC

Table 1 also shows the multivariate odds ratios for response to the NNSPC. Potential sociodemographic predictors were size of residential area, residential block, household structure, number of children, maternal age, and maternal labor force status. Children who lived in a smaller city, especially in the Hokuriku, Tokai, and Kyushu areas, or lived in households with a large number of children were more likely to respond to the survey than the respective reference group. Three-generation households were more likely to respond to the survey than families of parents and unmarried children only. Among potential maternal characteristics, mothers who were older (≥40 years) and who were non-workers were more likely to respond to the survey. In contrast, no independent associations were observed for child’s sex, child’s age, equivalent household expenditure, maternal educational attainment, or any paternal variable examined in relation to response to the survey.

### Results from a sub-analysis using income questionnaire

Of the 781 children who answered the income questionnaire of the 2015 CSLC, 544 children (69.7%) responded to the 2015 NNSPC. Table 2 shows the potential socioeconomic predictors of response among these responding subjects. Self-assessed living condition was significantly associated with response to the survey. Compared with households that assessed their living conditions as ‘normal’, those who assessed them as ‘very difficult’ were less likely to respond. This association remained after adjustment for potential confounders. On the other hand, we found no clear association between equivalent household annual income and response to the survey.

**Table 2.** Response rates and odds ratios for response to the 2015 National Nutrition Survey on Preschool Children, Japan, according to economic characteristics among children aged <6 years and their households who answered an income questionnaire

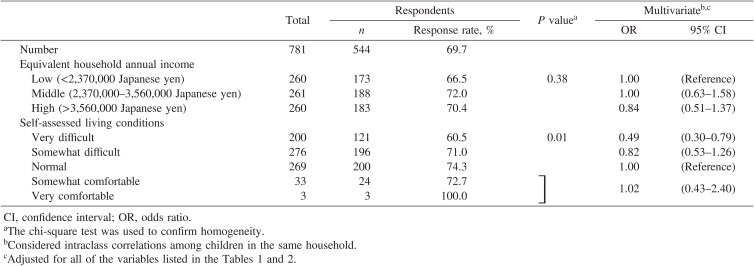

### Magnitude of bias due to selective response in survey estimates of the 2015 NNSPC

Table 3 shows the magnitude of bias in survey estimates of the 2015 NNSPC, taking account of missing data (ie, item non-response and survey non-response) through multiple imputation. Survey estimates examined between respondents before and after imputation for item non-response (columns A and B, respectively) were not substantially different, which suggests that bias due to item non-response was quite small. When we compared survey estimates of respondents (B) and non-respondents (C) after imputation to examine the magnitude of bias attributable to survey non-response, prevalence of overweight was higher in non-respondents (20.4%) than in respondents (14.8%). This led to biased distribution in overweight, and prevalence of overweight among respondents (A) was underestimated by 20.3% in comparison with the imputed survey estimates among potential survey participants (D). In addition, the number of children receiving treatment for constipation was underestimated by 25.4% of respondents, while the number with an irregular bedtime was overestimated by 15.0% of respondents. However, these absolute differences were negligible (−0.3% and 0.3%, respectively). For all other variables examined, the bias was much smaller (<5%).

**Table 3.** Magnitude of bias in survey estimates among respondents of the 2015 National Nutrition Survey on Preschool Children, taking account of missingness through multiple imputation^a^

	Respondents before imputation^b^ (A)	Potential survey participants after imputation^c^			

		Respondents (B)	Non-respondents (C)	All (D)	Estimated bias, %^d^
Items obtained from the 2015 NNSPC	(*n* = 3,426)	(*n* = 3,426)	(*n* = 1,917)	(*n* = 5,343)	
Child’s sex, boy, %	51.2	51.2	51.0	51.1	0.1
Child’s age, %					
0–1 year	31.8	31.8	31.4	31.7	0.4
2–3 years	33.7	33.7	34.2	33.9	−0.4
4–5 years	34.5	34.5	34.4	34.5	0.0
Birthweight, g	2998	2998	2991	2996	0.1
Birth length, cm	48.8	48.8	48.8	48.8	0.1
Gestational week, wk	38.7	38.7	38.7	38.7	0.0
Body weight, kg	13.9	13.7	13.8	13.8	1.1
Body height, cm	93.4	92.4	92.9	92.6	0.8
BMI, kg/m^2^	15.8	15.9	15.8	15.9	−0.1
Overweight, %^e^	13.4	14.8	20.4	16.8	−20.3
Birth order, first, %	45.6	45.6	45.9	45.7	−0.2
Place of day care					
Nurseries, yes, %	34.8	34.8	34.5	34.7	0.4
Kindergarten, yes, %	25.5	25.5	26.8	26.0	−1.8
ECEC, yes, %	4.5	4.5	5.0	4.7	−3.8
Home, yes, %	30.3	30.3	30.8	30.5	−0.6
History of food allergy, %	15.0	15.0	15.2	15.1	−0.4
Bowel movements per week, %					
Almost everyday	75.8	75.8	76.3	76.0	−0.3
4–5 times/wk	19.6	19.6	18.0	19.0	2.9
≤3 times/wk	3.7	3.7	3.7	3.7	−0.3
Under treatment	1.0	1.0	2.0	1.3	−25.4
Wake-up time on weekdays, %					
Before 7:00	46.5	46.6	45.9	46.3	0.5
7:00–7:59	43.9	43.9	44.3	44.0	−0.3
After 8:00	8.2	8.2	8.6	8.4	−1.4
Irregular	1.4	1.4	1.3	1.3	2.3
Bedtime on weekdays, %					
Before 21:00	28.3	28.3	29.4	28.7	−1.5
21:00–21:59	49.5	49.5	49.3	49.4	0.2
After 22:00	20.2	20.2	20.1	20.2	0.2
Irregularity	2.0	2.0	1.3	1.7	15.0
Maternal age, %					
<30 years	18.3	18.4	18.5	18.4	−0.5
30–39 years	64.2	64.2	62.7	63.6	0.9
≥40 years	17.5	17.5	18.8	17.9	−2.6
Maternal employment, yes, %	49.7	49.9	47.3	49.0	1.5
Self-assessed economic condition, %					
Difficult	37.5	37.5	38.0	37.7	−0.5
Normal	32.9	32.9	31.7	32.5	1.3
Comfortable	29.6	29.7	30.2	29.9	−0.8
Self-assessed time allowance, %					
Difficult	47.2	47.2	46.8	47.0	0.3
Normal	21.6	21.7	20.9	21.4	1.3
Comfortable	31.2	31.2	32.3	31.6	−1.3
Self-assessed overall living conditions, %					
Difficult	20.8	20.8	23.5	21.7	−4.5
Normal	37.8	37.8	37.4	37.6	0.4
Comfortable	41.5	41.5	39.1	40.6	2.0

CSLC, the Comprehensive Survey of Living Conditions; ECEC, certified centers for early childhood education and care; NNSPC, the National Nutrition Survey on Preschool Children.

^a^Values are means for continuous variables and percentage for categorical variables.

^b^Among 3,426 respondents to the NNSPC, data were missing for body weight (*n* = 265), body height (*n* = 378), BMI (*n* = 384), birthweight (*n* = 23), birth length (*n* = 64), gestational week (*n* = 60), place of day care (*n* = 7), history of food allergy (*n* = 21), bowel movements per week (*n* = 14), wake-up time (*n* = 12), bed time (*n* = 17), maternal age (*n* = 66), maternal employment (*n* = 80), self-assessed economic condition (*n* = 4), self-assessed time allowance (*n* = 2), and self-assessed overall living conditions (*n* = 2).

^c^Children aged <6 years and their households that answered the 2015 CSLC (= 3,426 respondents + 1,917 non-respondents).

^d^Computed, before rounding, by dividing the difference in the survey estimates between the respondents (A) and potential survey participants after imputation (D) by the imputed survey estimates in potential survey participants (D) and multiplying by 100.

^e^Overweight was defined according to the age- and sex-specific BMI reference data for Japanese children using the LMS method that are corresponded to BMI of 25 kg/m^2^ at 17.5 years of age.^25^

To examine the potential bias in associations between targeted outcomes and certain sociodemographic variables that significantly influence the response to the survey, we provisionally compared ORs of overweight, as an example of targeted outcome, by sociodemographic variables before and after imputation (Table 4). The prevalence of overweight substantially changed in all categories of sociodemographic variables examined after imputation. The difference in OR of overweight before and after imputation was broadly observed. This tendency was more marked in certain categories with low response rate, such as larger size of residential areas, single-parent families, and younger maternal age.

**Table 4.** Comparison of odds ratio of overweigh by sociodemographic characteristics estimated from observed and multiple imputation data

	Observed data of the NNSPC respondents^a^			Multiple imputation data^b^		
	
	Prevalence, %^c^	OR^d^	95% CI	Prevalence, %^c^	OR^d^	95% CI
Size of residential area						
Metropolitan area	11.2	1.00	(Reference)	15.5	1.00	(Reference)
City with population ≥150,000	14.2	1.31	(0.98–1.75)	17.2	1.12	(0.85–1.48)
City with population <150,000	14.2	1.27	(0.95–1.70)	17.6	1.17	(0.89–1.52)
Towns and villages	13.7	1.22	(0.79–1.89)	16.2	1.05	(0.71–1.55)
Household structure						
Parents and unmarried children only	13.1	1.00	(Reference)	16.6	1.00	(Reference)
Single parent and unmarried children only	5.3	0.41	(0.12–1.32)	13.3	0.93	(0.49–1.77)
Three-generation family	15.6	1.20	(0.90–1.61)	18.7	1.19	(0.93–1.53)
Others	14.0	0.94	(0.42–2.11)	17.8	1.00	(0.50, 1.97)
Maternal age						
<30 years	16.0	1.00	(Reference)	19.7	1.00	(Reference)
30–39 years	12.4	0.83	(0.63–1.08)	13.8	0.65	(0.55–0.78)
≥40 years	14.2	1.15	(0.81–1.64)	14.9	0.90	(0.69–1.18)
Maternal employment						
Yes	14.2	1.00	(Reference)	17.1	1.00	(Reference)
No	12.5	0.77	(0.62–0.96)	16.5	0.82	(0.68–0.99)

CI, confidence interval; CSLC, the Comprehensive Survey of Living Conditions; NNSPC, the National Nutrition Survey on Preschool Children; OR, odds ratio.

^a^Children aged <6 years and their households that answered both the 2015 CSLC and NNSPC (*n* = 3,426). Overweight was missing in 384 of the 3,426 respondents to the NNSPC in the observed data.

^b^Children aged <6 years and their households that answered the 2015 CSLC (*n* = 5,343).

^c^Overweight was defined according to the age- and sex-specific BMI reference data for Japanese children using the LMS method that are corresponded to BMI of 25 kg/m^2^ at 17.5 years of age.^25^

^d^Adjusted for residential blocks (Hokkaido and Tohoku; Kanto; Hokuriku and Tokai; Kinki; Chugoku and Shikoku; and Kyushu), child’s sex (boy or girl), child’s age (months), birthweight (g), birth length (cm), and gestational week (wk).

The statistics of the NNSPC published by the MHLW are generally estimated based on 3,871 NNSPC participants regardless of the availability of the CSLC data. Thus, we conducted the same analyses to examine the magnitude of bias in survey estimates among the NNSPC participants (*n* = 3,871) and the total survey participants of the CSLC and NNSPC (*n* = 5,788). The bias in point estimates for almost all variables, except for prevalence of overweight, in the NNSPC were small (eTable 1). Non-ignorable bias in ORs of overweight by sociodemographic variables was observed between before and after imputation (eTable 2).

## DISCUSSION

By linkage to national data of the CSLC, we found that overall response rate of the 2015 NNSPC was 64.1%, and that response to the survey varied according to several sociodemographic characteristics of households. Response rates were substantially lower in metropolitan areas, as well as among families with a small number of children, households with difficulty in living conditions, and mothers who were younger and workers. Although some biases were seen in survey estimates of the NNSPC, the magnitude of bias due to this selective response was generally small.

To our knowledge, this is the first study to clarify the response rate and sociodemographic factors influencing the response to the NNSPC through linkage to the CSLC. Comparison with other studies is, therefore, limited to other national surveys that were sampled from the CSLC. For example, a few studies have examined response rates of the National Health and Nutrition Survey (NHNS),^26^ an annual nationwide survey which assesses the health status, food and nutrient intake, and lifestyles of people living in Japan, using linkage to the CSLC.^27^^,^^28^ Response rates to the questionnaire surveys of the NHNS from 2003 to 2007 were 59.4–66.6% for the dietary survey and 59.2–67.9% for the lifestyle questionnaire.^27^ A similar but slightly lower response rate (56.9%) was reported in the 2011 NHNS.^28^ Respondents of the NHNS were more likely to be women and older.^27^^,^^28^ In addition, response rates were higher in households with a large number of family members, three-generation families, and self-employed families, and lower in single-parent families and employed families.^27^ In a comparison study with the Population Census in 2010, the NHNS sample had a lower proportion of individuals living in a metropolitan area (Kanto).^29^ Although direct comparison is hindered by differences in survey methods, target population, and survey year, the response rate of the NNSPC (64.1%) was comparable to those for the NHNS and the results of this study are in accordance with the main tendencies of the findings of the NHNS: responses were associated with smaller cities, more family members, particularly children, a three-generation family structure, and women (mothers) with older age. These findings from the NNSPC and NHNS suggest that residential area, number of household members, family structure, sex, and age are common predictors of a response to national surveys in Japan.

Partially consistent with previous studies,^5^^–^^12^ we observed that response to the survey was related with age and occupation. Of note, however, these relations were observed only in factors derived from mothers, not fathers. Because the questionnaires were answered mainly by mothers (95.7%), response status might be more strongly affected by maternal factors. It is well-known that maternal socioeconomic factors and their health behaviors are associated with child’s diet quality and general health behaviors.^30^^–^^32^ The respondents of the NNSPC might be already biased according to maternal characteristics. Although bias caused by selective response in ORs of overweight in the NNSPC was observed, we still do not know the magnitude of bias in estimates of the other targeted outcomes across categories of maternal socioeconomic status. Further investigations of effects of bias by maternal and household sociodemographic characteristics is required to assess associations between variables more precisely. Moreover, any interpretation of survey findings on the relationships between maternal influence and child’s diet require the closest attention.

The relation of some sociodemographic characteristics and response rates might be related to survey method. We speculated that a home-visit survey with a drop-off self-administered questionnaire as used in the NNSPC may involve more difficulty in contacting potential participants for survey invitation and/or data collection, particularly in areas with a large number of housing complexes, households with a small number of family members, and employees with long commutes, with consequent low response. Improving response rates therefore requires efforts to increase rates of contact with potential participants, and will in turn reduce selection bias. An increasing number of studies have investigated how to improve response rates in health surveys.^33^^–^^36^ Several studies examined possible reasons for non-participation in health surveys. Common answers among non-respondents were lack of time, unsuitable timing, increased number of survey requests, little personal benefit, and not considered relevant.^33^^–^^35^ A systematic review listed example strategies that might increase response to postal and electronic questionnaires, including monetary incentives, a more interesting questionnaire topic, pre-notification, follow-up contact, and shorter questionnaires.^36^ Although the NNSPC uses a different survey method from the result of this systematic review, at least some of them would also likely be effective in increasing response rates in the NNSPC. Additional research on reasons for non-participation and ways to enhance motivation to participate in future surveys in Japan are warranted.

Missing data due to selected survey participation is a serious challenge and a threat to the validity of survey statistics. In the present study, we used a multiple imputation approach and conducted comparison between respondents, non-respondents, and potential survey participants after imputation to estimate magnitude of the bias in the survey estimates of the key variables. Although the response rate varied according to sociodemographic characteristics, bias in population estimates seemed quite small in most of the variables examined. In contrast, bias caused by selective response in prevalence and ORs of overweight was observed. These results suggest that the OR could be biased when the following two conditions are met simultaneously: 1) the prevalence of an outcome (eg, overweight) is substantially different between respondents and non-respondents, and 2) the response rate is substantially different between categories of a factor (eg, maternal age categories). An association analysis using data with low response rate should be carefully interpreted considering these conditions.

Among the strengths of our present study are its large nationwide sample of Japanese preschool children and their households from the general population. Further, linkage to the CSLC enabled us to extract a wide range of information on the sociodemographic and economic variables of households which we could then use to compare subject characteristics between respondents and non-respondents to the NNSPC. In addition, the use of the multiple imputation approach allowed us to maximize the available information through plausible replacement values for missing data and to evaluate the direction and magnitude of possible bias caused by selective response in survey estimates of the 2015 NNSPC.

Several limitations also need to be considered when interpreting the study findings. First, potential survey participants in the NNSPC (ie, respondents of the CSLC who were randomly sampled from the National Census in 2010) might already be inconsistent with the general population, given that the response rate of the 2015 CSLC was 78.5%.^21^ Second, we were limited in our comparison of sociodemographic factors between respondents and non-respondents. Although many studies have reported relationships among health status, lifestyle, and mortality,^5^^,^^9^^–^^13^ as well as of sociodemographic characteristics with response status, no such information was available in the CSLC we used because it was conducted as a small-scale survey in 2015. Third, there were 445 children who answered the NNSPC but were not linked with the CSLC, which in theory at least should not happen, considering that the CSLC provides sampling frames for the NNSPC. A few of these children were newly invited to the NNSPC because they had moved into the survey area during the survey period, and at least 236 children could not be linked to the CSLC because of inconsistent or missing information for one of the key variables used to identify individuals between the two surveys (ie, birth year, *n* = 50; birth month, *n* = 21; and sex, *n* = 165). We do not know the exact proportion of these cases because of a lack of information and the expiration of the retention period of the originally answered questionnaires in the MHLW. In addition, subjects who had data for the NNSPC only were excluded from the present analysis, which might have led to further bias. This was done because some of the sociodemographic variables examined here were not available in the NNSPC, which made it difficult to compare the background characteristics of subjects with and without the CSLC data. Nevertheless, survey estimates of the 2015 NNSPC were not substantially affected regardless of the inclusion of NNSPC-only cases in multiple imputation. Fourth, the variables examined for the magnitude of bias were limited to common question items observed in questionnaires for 0–1-year-olds and 2–5-year-olds in the 2015NNSPC. Therefore, future studies aiming to more precisely assess diet-health relationships among young children should first confirm potential bias in estimates for other variables. Fifth, overweight, as an example of targeted outcome, was defined using BMI calculated from guardian-reported information, which might be biased. More importantly, BMI is based on only weight and height, both of which greatly change during growth and development. Finally, although we controlled for a wide range of potential confounders in multivariate logistic regression analysis, we cannot rule out the possibility of unmeasured or residual confounding, which is inherent to observational studies.

In conclusion, this study of a large sample of Japanese preschool children and their households showed that response to the survey varied according to sociodemographic characteristics, such as residential size and area, family structure, household economic status, and maternal age and labor force status. Allowing that certain subgroups are underrepresented, the general validity of survey estimates of the 2015 NNSPC, with the exception of prevalence of overweight, do not appear to be prejudiced by the bias produced by non-response. In addition to further improvement of response rate to the NNSPC, more insight into the effects of selective response is required to assess diet-health relationships among young children more precisely.

## APPENDIX A. SUPPLEMENTARY DATA

eTable 1. Magnitude of bias in survey estimates among participants that answered either or both the CSLC and NNSPC, taking account of missingness through multiple imputation^a^

eTable 2. Comparison of odds ratio of overweigh by sociodemographic characteristics estimated from observed and multiple imputation data
