# Supplementary material for: Sociodemographic Factors Influenced Response to the 2015 National Nutrition Survey on Preschool Children: Results From Linkage With the Comprehensive Survey of Living Conditions
Source: J Epidemiol. 2020 Feb 5;30(2):74–83. doi: 10.2188/jea.JE20180176 (PMC6949187; doi:10.2188/jea.JE20180176)
Supplement: Supplementary file 1 [file je-30-074-s001.pdf]

eTable 1. Magnitude of bias in survey estimates among participants that answered either or both the CSLC and NNSPC, taking account of missingness through multiple imputation[a]

Items obtained from the 2015 NNSPC	NNSPC participants before imputation[b] (A) (n=3,871)	Potential survey participants after imputation[c] NNSPC participants (B) (n=3,871)	Non-participants[d] (C) (n=1,917)	Total (D) (n=5,788)	Estimated bias (%)[e]
Child's sex, boy, %	51.2	51.5	51.0	51.3	-0.3
Child's age %					
0–1 year	32.2	32.2	31.4	32.0	0.9
2–3 years	33.6	33.6	34.2	33.8	-0.5
4–5 years	34.1	34.1	34.4	34.2	-0.3
Birthweight, g	3000	3000	2991	2997	0.1
Birth length, cm	48.9	48.8	48.8	48.8	0.1
Gestational week, wk	38.7	38.7	38.7	38.7	0.0
Body weight, kg	13.9	13.7	13.8	13.7	1.0
Body height, cm	93.3	92.3	92.9	92.5	0.9
BMI, kg/m^2	15.9	15.9	15.8	15.9	-0.1
Overweight, %[f]	11.9	15.1	20.4	16.8	-29.4
Birth order, first, %	45.6	45.6	45.9	45.7	-0.2
Place of day care					
Nurseries, yes, %	35.4	35.4	34.5	35.1	0.9
Kindergarten, yes, %	24.6	24.6	26.8	25.3	-2.9
ECEC, yes, %	4.8	4.8	5.0	4.9	-1.2
Home, yes, %	30.1	30.1	30.8	30.3	-0.8
History of food allergy, %	14.9	14.9	15.2	15.0	-0.7
Bowel movements per week, %					
Almost everyday	76.5	76.5	76.3	76.4	0.1
4–5 times/wk	18.9	18.9	18.0	18.6	1.6
≤3 times/wk	3.6	3.6	3.7	3.6	-0.8
Under treatment	1.0	1.0	2.0	1.3	-22.4
Wake-up time on weekdays, %					
Before 7:00	46.7	46.7	45.9	46.4	0.5
7:00–7:59	43.7	43.7	44.3	43.9	-0.4
After 8:00	8.3	8.3	8.6	8.4	-1.1
Irregular	1.4	1.4	1.3	1.4	1.5
Bedtime on weekdays, %					
Before 21:00	28.5	28.5	29.4	28.8	-1.1
21:00–21:59	49.0	49.0	49.3	49.1	-0.1
After 22:00	20.6	20.6	20.1	20.4	0.8
Irregularity	2.0	2.0	1.3	1.8	13.1
Maternal age, %					
<30 years	19.3	19.3	18.5	19.0	1.4

30–39 years	63.3	63.2	62.7	63.0	0.3
≥40 years	17.5	17.5	18.8	17.9	-2.6
Maternal employment, yes, %	50.4	50.7	47.3	49.6	1.7
Self-assessed economic condition, %					
Difficult	37.6	37.6	38.0	37.8	-0.3
Normal	33.0	33.0	31.7	32.6	1.4
Comfortable	29.3	29.4	30.2	29.7	-1.1
Self-assessed time allowance, %					
Difficult	47.4	47.4	46.8	47.2	0.4
Normal	21.5	21.5	20.9	21.3	0.9
Comfortable	31.1	31.1	32.3	31.5	-1.3
Self-assessed overall living conditions, %					
Difficult	21.1	21.1	23.5	21.9	-3.6
Normal	37.5	37.5	37.4	37.5	0.1
Comfortable	41.4	41.4	39.1	40.6	1.8

CSLC, the Comprehensive Survey of Living Conditions; ECEC, certified centers for early childhood education and care; NNSPC, the National Nutrition Survey on Preschool Children.

[a] Values are means for continuous variables and percentage for categorical variables.

[b] Children aged <6 years and their households that answered the 2015 NNSPC regardless of participation in the 2015 CSLC. Among 3,871 participants to the NNSPC, data were missing for body weight (n=303), body height (n=439), BMI (n=445), birthweight (n=28), birth length (n=81), gestational week (n=71), place of day care (n=8), history of food allergy (n=24), bowel movements per week (n=15), wake-up time (n=15), bed time (n=23), maternal age (n=83), maternal employment (n=97), self-assessed economic condition (n=5), self-assessed time allowance (n=2), and self-assessed overall living condition (n=2).

[c] Children aged <6 years and their households that answered either or both the CSLC and NNSPC (= 3,426 participants with CSLC + 445 participants without CSLC + 1,917 non-participants) .

[d] Column C shows the same data as that in Table 3.

[e] Computed, before rounding, by dividing the difference in the survey estimates between the NNSPC participants (A) and total survey participants (D) after imputation by the imputed survey estimates in total survey participants (D) and multiplying by 100.

[f] Overweight was defined according to the age- and sex-specific BMI reference data for Japanese children using the LMS method that are corresponded to BMI of 25 kg/m^2 at 17.5 years of age.[25]

eTable 2. Comparison of odds ratio of overweigh by sociodemographic characteristics estimated from observed and multiple imputation data

	Observed data of the NNSPC participants[a]			Multiple imputation data[b]		
	Prevalence, %[c]	OR[d]	95% CI	Prevalence, %[c]	OR[d]	95% CI
Size of residential area						
Metropolitan area	11.2	1.00	(Reference)	15.5	1.00	(Reference)
City with population ≥150,000	14.2	1.31	(0.98–1.75)	17.2	1.12	(0.86–1.46)
City with population <150,000	14.2	1.27	(0.95–1.70)	17.7	1.17	(0.90–1.51)
Towns and villages	13.7	1.22	(0.79–1.89)	16.3	1.05	(0.71–1.54)
Household structure						
Parents and unmarried children only	13.1	1.00	(Reference)	16.6	1.00	(Reference)
Single parent and unmarried children only	5.3	0.41	(0.12–1.32)	13.6	0.95	(0.51–1.75)
Three-generation family	15.6	1.20	(0.90–1.61)	18.8	1.20	(0.93–1.54)
Others	1.8	0.94	(0.42–2.11)	17.8	0.99	(0.51–1.90)
Maternal age						
<30 years	13.6	1.00	(Reference)	19.9	1.00	(Reference)
30–39 years	11.1	0.83	(0.63–1.08)	14.0	0.66	(0.55–0.77)
≥40 years	12.5	1.15	(0.81–1.64)	14.6	0.87	(0.67–1.12)
Maternal employment						
Yes	12.4	1.00	(Reference)	17.2	1.00	(Reference)
No	11.3	0.77	(0.62–0.96)	16.5	0.80	(0.67–0.96)

CI, confidence interval; CSLC, the Comprehensive Survey of Living Conditions; NNSPC, the National Nutrition Survey on Preschool Children; OR, odds ratio.

[a] Children aged <6 years and their households that answered the 2015 NNSPC regardless of participation in the 2015 CSLC (n=3,871). Overweight was missing in 445 of the 3,871 participants to the NNSPC in the observed data.

[b] Children aged <6 years and their households that answered either or both the CSLC and NNSPC (n=5,788).

[c] Overweight was defined according to the age- and sex-specific BMI reference data for Japanese children using the LMS method that are corresponded to BMI of 25 kg/m^2 at 17.5 years of age.[25]

[d] Adjusted for residential blocks (Hokkaido and Tohoku; Kanto; Hokuriku and Tokai; Kinki; Chugoku and Shikoku; and Kyushu), child’s sex (boy or girl), child’s age (months), birthweight (g), birth length (cm), and gestational week (wk).
